# Dynamic comparison of PET imaging performance between state-of-the-art ToF-PET/CT and ToF-PET/MR scanners

**DOI:** 10.1186/2197-7364-1-S1-A75

**Published:** 2014-07-29

**Authors:** Gaspar Delso, Tim Deller, Mehdi Khalighi, Patrick Veit-Haibach, Gustav von Schulthess

**Affiliations:** GE Healthcare, Kragujevac, Switzerland; University Hospital of Zurich, Kragujevac, Switzerland

The goal of the present work was to determine the potential for dose reduction in a new clinical ToF-PET/MR scanner. This was achieved by means of long dynamic phantom acquisitions designed to provide a fair comparison of image quality and lesion detectability, as a function of activity, between the new PET/MR system and a state-of-the art PET/CT.

A NEMA body phantom was scanned, first on a GE ToF-PET/CT (D690) and a ToF-PET/MR. The phantom was filled with 130 MBq ^18^F with 1:4 ratio between background and the four smaller spheres. The larger spheres and lung insert were filled with regular water. In each case a three-hour data acquisition was stored as list-mode.

The data were unlisted to create 2-minute sinograms every 5 minutes. PET/CT was reconstructed using VUEPointFX, 4it24sub, 256x256pix, 40cm FOV, no SharpIR, no axial, 2mm transaxial filter. PET/MR used VUEPointFX, 4it28sub, 256x256pix, 40cm FOV, no SharpIR, “light” axial, 2mm transaxial filter.

ROIs were placed in the slice intersecting the spheres center (background, lung insert, biggest and smallest hot spheres). Plots of ROI statistics were used to evaluate IQ vs. activity. The visual impression is of better image quality, due to the smaller crystal size (3.95x5.3x25mm^3^ vs. 4.2x6.3x25mm^3^) and larger axial FOV (250mm vs. 157mm) of the PET/MR. The average reconstructed intensity is equivalent for the hot spheres and insert, background intensity being 3% larger in PET/CT. No significant differences in intensity standard deviation were found for any ROI.

The new PET/MR scanner provides equivalent detectability with larger FOV and higher spatial resolution. An equivalent dose reduction to oncology patients could be implemented, exploiting the increased FOV.

**Figure 1:**
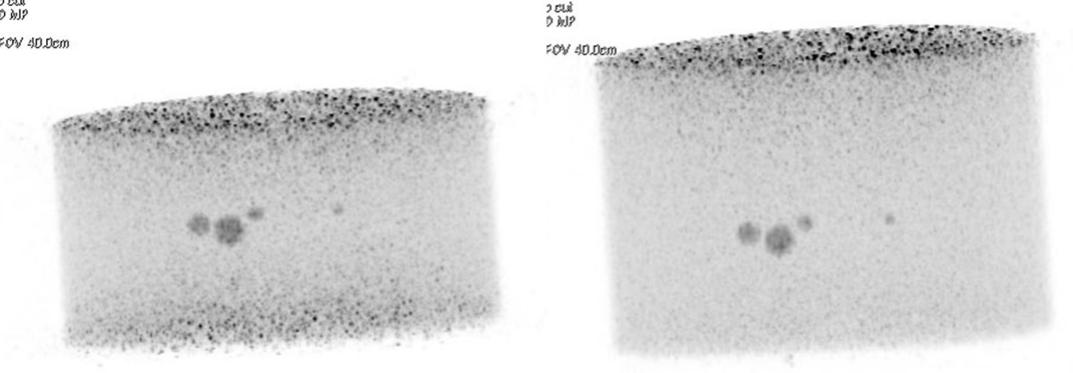
Three-dimensional maximum-intensity projection of equivalent frames (approx. 80 MBq) in the PET/CT (left) and PET/MR scanner (right)

